# Medical Deontology, or the Duties and Rights of Medical Men in the Present State of Civilisation

**Published:** 1846-10-01

**Authors:** 


### Deontologie Medicale, ou des Devoirs et des Droits des Medecins, dans l'Etat actuel de la Civilisation. Par le Docteur *Max. Simon.* Octavo, pp. 567. Paris, 1845. Bailliere.

### Medical Deontology, or the Duties and Rights of Medical Men in the present State of Civilisation. By Dr. *Max. Simon.*

It was only in our last number that the following passage occurs at the close of an article upon Military Punishments: "Surely there is no occupation, in which medical men can more profitably fulfill their beneficent *mission,* than in using all their professional influence and authority to plead the cause of the suffering and oppressed against the heartless neglect or tyrannical severity of irresponsible taskmasters. Of recent years, the fruits of enlightened medical philanthropy have been gloriously displayed in the changes already effected in our prisons and lunatic asylums, &c., and in the improved general œconomic treatment of our pauper population. May the same good spirit continue to animate every member of the profession, in whatever sphere he may be placed! It is thus only that our calling may justly claim to itself the proud distinction, that has been assigned to it by the eloquent orator of antiquity, of being 'an art almost divine.'"

When we wrote these words, we did not expect to have so early an opportunity of directing the attention of our readers to the general question of Medical Deontology,* or, in other words, that of the Rights and Duties of our profession. Dr. Simon's work had indeed been published several months before; but it is only within the last few weeks that it has been brought under our notice. The perusal of it, we rejoice to say, has given us very great satisfaction. Everywhere it breathes a spirit of high-toned moral feeling, and dignified independence of thought. May its influence be felt and appreciated by his countrymen! To us it will be a source of very sincere pleasure if the extracts, which we propose to select from its pages, have the effect of introducing it to the notice of the British medical public, and of its merits being acknowledged by our press. Much honour is due to the literature of France that, amid the general apathy and indifference of medical men of all countries, in the present century, to the intimate connection between their very responsible calling and the exercise of the highest social virtues, one of her sons has not been ashamed to step forward with the bold avowal and manly vindication of principles, which, although they may be regarded as Utopian or Puritanical by many, cannot but actuate every truly great and good physician in his intercourse with the sick and afflicted of mankind. He will ever remember that he has other duties, besides those of studying the physical effects of disease, or of prescribing remedies for its relief; great and pressing duties which he owes to himself, to society, and to his God. It is the sin of the present age to view everything with a sort of materialising and mercantile spirit, to estimate the value of an object only by its immediate results, and by the effects which it sensibly and visibly produces. It is an age of facts rather than of principles, of experiment rather than of reflection. How strikingly is this the case in all that appertains to the science and art of Medicine. Every year, nay, every month or every week, brings forward its new remedy, its novel discovery. The journals teem with practical *facts*—so, at least, are they supposed to be, and so are they termed. It is almost surprising that anything now remains to be found out, considering the abundance of successful or infallible remedies or modes of treatment for almost every disease. Then again in Physiology, every thing is determined by positive experiments on living animals; and, when so determined, it is asked how can the conclusions, so derived, fail to be correct? It may be so; but we should like to be told, how comes it to pass that there is so much discrepancy and downright contrariety of statements and conclusions among different physiological observers? The question is puzzling; where is the solution to be found? What is to be done? If we might presume to answer, we should say; watch more, work less; interrogate nature by long and patient observation, rather than by rough force or cruel compulsion. Are we not told that not even a sparrow falls

* This term was first used by Bentham to denote the science of Ethics. It is derived from δεον *quod decet, æquum,* and λογος *sermo.*

to the ground without the knowledge of Him who made it? What then shall we say of the shambles of the modern vivisector? But it will not do to digress too far from our more immediate subject.

We have said that the medical men of the present day have their attention directed almost exclusively to the sensible phenomena, the practical details of their profession, and thus overlook, and perhaps have never so much as thought of, any other duties that may be required of them. Medicine, it would seem, is not now regarded as a high and holy pursuit, that ought to elevate the thoughts and spiritualise the feelings; it is a mere vocation by which a man earns his bread. "Healing the sick" is no longer a sacred calling; it is a trade, a mercantile occupation. Hence the numerous and varied means by which its votaries seek for distinction and repute. One man succeeds by keeping an open shop and dealing in perfumery and physic; another attracts his neighbours by the brilliant-coloured light over his door: a third is active in parochial matters, and acquires the name of a clever, smart man of business; a fourth trusts chiefly to his carriage to drive him into practice; a fifth communicates the results of his extraordinary success in the treatment of certain diseases to the columns of a journal, or gets a puff into a newspaper; while a sixth makes a hit by a well-timed popular work, dressed out, it may be, in the most attractive style. All is done for effect, to catch applause, to build a fortune. We say this, not so much in the spirit of censure as in that of regret;—regret at witnessing so noble a profession as ours degraded to the level of a common trade. This is assuredly not as it ought to be; but that such is really the case, will not be denied by many. And what is the cause of this state of things?—simply and solely this; forgetfulness or disregard of the real extent and degree of the *duties* which the pursuit of the medical profession involves. But then, it will be asked, what are these duties that are spoken of? Sydenham suggests them in these memorable words:—

"Whoever applies himself to Medicine should seriously consider the following things. In the *first* place, that he must one day render to the Supreme Judge an account of the lives of those who have been entrusted to his care. *Secondly*, that whatever skill or knowledge, under divine blessing, he has acquired, ought primarily to be directed to advance the glory of God and the welfare of mankind; it is a disgrace to make these heavenly gifts subservient merely to the ends of avarice or ambition. *Thirdly*, that he has undertaken the care of one who is neither ignoble nor to be contemned: in order that he may estimate the value of a human being aright, let him remember that the only-begotten Son of God became man, and ennobled the nature which He assumed. *Lastly*, that he (the physician) himself is not exempt from the common lot of mankind, but is subject to the same laws of mortality, the same accidents and the same sorrows as others; therefore let him, fellow-sufferer (*ομοιοπαθης*) as he is, with greater diligence and love seek to relieve the sick and the afflicted."

Now the question comes to be,—supposing that we are willing to take our own great countryman as a teacher of professional ethics—are these the motives that actuate medical men in the present day? Let us hope that they are so; but are we warranted in entertaining such a hope? Without prying into the recesses of each other's hearts, may we not fairly and in the spirit of perfect charity judge, to a certain degree at least, by the outward acts and manifestations of conduct? Do any medical books or lectures, in the present day, ever breathe so hallowed and so solemn a spirit as the words which we have just quoted? Have not some of our recent works on Physiology been polluted with open indecencies, and has there not been an air of infidel presumption cast around not a few of the vaunted discoveries of modern times? Go to our very temples of medical science, and listen to the senators of the profession, clothed in their robes of authority, and seated, it may be, in their chairs of state. Has not the hall of one of our Colleges, within the last twenty years, been made the theatre where atheistic scepticism and bold impiety have been proclaimed under the garb of scientific exposition, and where the most injurious calumnies have been uttered against honorable men, who had no opportunity of reply?—and have we not heard of the learned fellows of another College now listening with a most edifying patience to the frippery of a semi-classical semi-fashionable gossip respecting the deaths of distinguished characters, and then sanctioning by their presence the advocacy of the juggleries of mesmeric artifice? Is it in such ways as these, that the established authorities of our profession seek to elevate its importance or enhance its utility? True; the sins or follies of one or two individuals are not to be charged against an entire body; but then, has either of the public bodies referred to disavowed all sympathy in such proceedings, or protested against the desecration of a noble science by such sillinesses on the one hand, and such profligacies on the other? Again, what has been done by these institutions—and here our remarks apply more especially to the College of Physicians—to which so much power and so many privileges have been granted, to promote the cause, we shall not say of the profession, but of medical philanthropy or beneficence throughout our land? Has it been to them that society is indebted for any of those ameliorations, or even for any suggestion touching those ameliorations, in the hygienic condition of our working classes, that are now happily on foot? Has any thing been done by them to enlighten and direct the government in enquiries respecting the state of our prisons, penitentiaries, or workhouses; the influence of trades and occupations on health; the effects of different sorts of food and of different geographical localities on the same important subject; the spread of contagious, and the diffusion of epidemic diseases; the intricate question of quarantine; the establishment of medical missions in countries where human beings are left to pine in wretchedness and suffering from want of professional relief?—not to mention a variety of other kindred subjects, all of which ought to be viewed as belonging to the domain of the science of medicine, and the investigation of which rightly appertains to one at least of our public medical establishments. Hitherto, little or nothing has been achieved in this field of most useful labour, save by individual exertion and zeal. We must indeed except from this rebuke the conduct of our Army and Navy Medical Boards; for to them, under the admirable superintendence of their present most enlightened and conscientious directors, is the profession indebted for numerous and very valuable acquisitions to medical knowledge, which no private individual could have effected. Can we say as much for the College of Physicians? The question constantly recurs to one's mind, what has it, in its corporate capacity, done for science or humanity? Even when directly appealed to and consulted by government on matters immediately appertaining to the health of the community, the conduct of its leading men, viewing it as a medical cabinet or institute, has seldom commanded the confidence either of the profession or of the public. Witness, for example, the doings of the Board of Health that was appointed on the approach and during the invasion of the Cholera. It is unnecessary to multiply instances of the utter inefficiency of this College; its very existence is more negative than positive. How shall we explain or account for such a state of things?—the answer is at hand; we have already spoken it. Those, who have had authority in its affairs, have either never duly considered, in its full and simple bearings, the solemn subject of Medical Deontology (to use our author's term) as it has been so emphatically laid by him, whose very name gives lustre to their college, or—painful as the assertion of the alternative is—they have wilfully and unworthily sacrificed principle and duty to the claims of selfish or corporate interest. Let us in charity believe that their error has proceeded rather from ignorance and want of due reflection, than from disregard of obvious moral requirements. The great and paramount question that we ought all to put to ourselves, whether as individuals or as members of a public body, is sternly and simply this; what is our *moral* duty? It is not what do we owe to such and such a one for having promoted our views, or coincided with our opinions? nor yet, what will best promote the secular interests or respectability of our party or our college? The only infallible and unerring guide of conduct is to "*do justly,* love mercy, and walk humbly before God." Hear what our author says in the admirable Introduction with which he prefaces his work. After alluding to the utter inefficacy of mere statutary enactments to teach medical men their duty (morally, not technically, be it remembered) under various trying emergencies, temptations, and harassing perplexities of their profession, he says:

"To the man who refers to God only, in his decisions and actions upon the subject of a thing so precious as life, the thought of God should ever be present; a philosophy, in which the sap of this fertilizing thought does not circulate, must be incapable of being the constant guide to the physician, amidst the numerous obstacles he encounters in the exercise of his profession. The study of medicine mounts up to God by the sympathy which the sight of suffering awakens in us; but science as it is of so lofty an origin, it does not completely do its work, save on the condition of requiring from Charity her love and her devotedness. The physician, who submits his conscience to the light of this elevated philosophy, may err; but his faults will only be imputable to the imperfections of his science. Comprehending the dignity of human nature and the deep signification of life, he devotes himself entirely to the study of a science, which may exercise so decided an influence over the individual destiny of men. Prudent, circumspect, he will not be found carelessly to adopt those premature theories, which sometimes pass over a generation like a fatal epidemic. In cases where theory and experience deny him positive instruction, he will confine himself within the line of a sage expectation. To whatever rank of society he belongs, the suffering man will be his brother by the double fraternity of pain and hope; through the most repulsive rags of misery, he will recognize in him the indelible character of his heavenly origin, and will proffer to him with a tender solicitude the most devoted attentions. Kind and considerate to all, he will not avail himself of amenity of language as a means of acquiring wealth or distinction. He will know that suffering spiritualizes man, if we may so speak, and imparts momentarily even to the most rugged natures a delicacy of sensibility, which a *brusquerie*

of manner deeply offends. To the humble pallet of the poor, as to the perfumed couch of the rich, he will bring the same gentleness, the same affability; and thus, while he discharges a hard duty, he also renders more sure the action of those curative means which science has prescribed. In a word, in whatever situation the physician is placed by the exigencies of his profession, he has imperative duties to fulfill, both towards himself and towards society, and it is from his conscience alone that he can acquire the light he needs to direct him with safety.

"But Conscience abandoned to its sole inspirations may stumble in those dark paths, in which she should guide us; she is accessible to all the passions, she has her caprices, like every power which is not attached to something fixed and immoveable. To find then a more sure guide, we must rise still higher, even to Christianity itself, which provides infallible instructions for all the circumstances of life, to Christianity, whose doctrine summed up in a single word, Charity, allies itself so wonderfully with a science, whose chief end is the relief of human suffering."

There is much sound philosophy as well as virtuous feeling in the following remarks on the powerful influence, either for good or for evil, which a medical man may have on the future welfare, no less than upon the present comfort, of the poor creatures that commit themselves to his care. It would serve to exalt the character of our profession not a little, if this subject were more frequently and more seriously considered than there is reason to believe that it is by those whom it concerns.

"In great cities, in manufacturing towns, where the enticement of higher wages or the seductions of pleasure tend more and more to concentrate population, the physician is called to exercise a powerful influence over the welfare of men. He may spread around him most fertile ideas; speaking by turns the language of science, of reason, of morality, knowing by a wise and prudent intimidation how to render the sufferings of disease conducive to a salutary reform, he may have it in his power, at the same time, to bring back to virtue and restore to health a multitude of unfortunate beings who abide in ignorance, misery and crime. There is between these three elements, which a gloomy etiology so often shows us in the pathology of misfortune, a mysterious affinity which reveals itself to the least attentive observer. Disease, in the thousand forms it assumes, is in many cases the sinister expression of this degradation, or at least of this incomplete development of man. Suffering, by suspending in a moment the illusions of passion, causing the deceptive mirage of pleasure to cease, and restoring to reason the freedom of its judgment, admirably prepares the mind for lessons of virtue. A magnificent apostleship is here offered to the physician, whose generous heart ought not to be a stranger to any of the miseries of mankind; and, while his hand places over the wound of the body the *dictamnus* of science, he may not unfrequently awaken in the deadened soul the feeling of the dignity of human nature. Not that we pretend that the physician should here usurp the office with which the priest is clothed in Christian societies; his work is pre-eminently a work of science, which he ought beyond every other to strive to accomplish; but if, in the accomplishment of this work, he ought constantly to be inspired with the sentiment of a tender sympathy, with a devoted charity, it is impossible for the man who has comprehended the importance of this mission not to rise up to the source of the evil, the effects of which he combats in the fatal maladies with which the body is affected. In how many instances is the prophylaxy nothing else than the destruction of vice, under whose influence the most robust constitutions are seen to bend? In using an instrument towards the instruction of morality, we not only do not go beyond the limits of science and art, understood in their real relations, but we should evidently fail in our object, if we did not reach the first cause of those organic or functional disorders which we have under our eyes. Many physicians before us have alluded to this relation between medicine and morality, and have endeavoured to shew how one might become a useful auxiliary to the other. Although a similar attempt, renewed in the present day, and by an author who has scarcely any other claim to the attention of those whom he addresses than the purity of his intentions, may be a little venturesome, we shall boldly enter this path, convinced as we are that the physician, who walks therein, resting upon sound doctrine, may serve one of the gravest interests of society."

M. Simon, evidently deeply impressed himself with the responsibility of the mission which a medical man voluntarily takes upon himself, takes every opportunity of urging upon his younger readers the necessity, nay the policy, of their being guided upon all occasions, not by love of fame, wealth, or distinction, but solely and entirely by high moral principle.

"In all professional careers, which suppose in those who have embraced them a high degree of intellectual development, the only rule of conduct worthy of the lofty nature of man is the abstract idea of duty; an idea that is practically embodied in a devotedness to the common weal. True, the man of intellect requires to be housed, fed, and clothed as well as the mechanic. Nay, the physical wants of life seem to increase with the degree of intellectual development; and the question therefore comes to be, how is this severe moral law, which we maintain is especially obligatory on the professional man, to be reconciled with the legitimate satisfaction of these wants? We answer—by another law, the truth of whose existence is daily exemplified in the movement of human affairs; viz. the almost constant coincidence of private advantage with public good. And even should it be the case that he, who devotes himself to the benefit of his fellow-creatures, finds himself deprived of that position which his services entitle him to expect, shall he not find some compensation for his trials in the secret joys of an approving conscience? In the noble inscription which Fabricius d'Aquapendente had placed over the door of his study—*lucri neglecti lucrum*—we may read the satisfaction of one who had found happiness in doing good."

These are noble sentiments, and do honour to our author's heart. Let them not be looked upon as the mere fancies of Utopian benevolence. There is more worldly wisdom, than most men of the world will admit, in the reflection that "there is that scattereth and yet increaseth; and there is that withholdeth more than is meet, but it tendeth to poverty."

The grand object of the Second Chapter, which treats of the "influence of medical studies and of the habitual spectacle of suffering upon the *morale* of medical men," is to guard them against the materialism of thought and feeling with which our profession has been so often reproached. From having so much to do with the mere machinery of life, the mind of the young enquirer is apt to identify the cause of a function with the organ that performs it; and thus, upon all occasions, to associate the existence of a vital principle with the presence of a certain mechanism and of certain chemical changes that are continually going on within the living body. The results too of the study of Pathological Anatomy on the one hand, by shewing the frequent, although certainly not the constant, connection between abnormal structure and disturbed or perverted physiological development, and, on the other hand, of Comparative Anatomy, unfolding as this science does the relations between organization and the gradual manifestations of life, have tended not a little to this contracted state of mind, that would in all cases limit the investigation of truth to those facts only that are discoverable by the scalpel, microscope or test-tube. It is the duty of the professor or public instructor to seize, every now and then, a favourable opportunity to spiritualise the facts and phenomena of physical research, and to divert the thoughts of the young enquirer from the mere materialism of the science which he is teaching, by lifting them to the contemplation of moral and religious truth.*

"Galen, at the close of his work on Anatomy, struck with the marvellous harmony of design that pervades the complicated mechanism of organization, exclaims that his book is a hymn to the Divinity; and it is told of M. Biot that, when relating the wonderful discoveries of Newton, he stopped short in the midst of his discourse, and wept tears of admiration. Unfortunately all medical men are not capable of such elevation of thought and sentiment; whether it be that the materialism of their pursuits has contracted to them the perspectives of life, or that they do not feel any longing desire to pass the limits of cold analysis. It cannot be doubted but that, upon minds that are constituted so, the exclusive study of the material side of life, the observation of the phenomena of the suffer-

---

* The last Hunterian Oration afforded a melancholy instance of the evil effects which a neglect of the principles here inculcated will almost inevitably produce, even in men of high literary and professional attainments. He, who thinks and talks lightly of those subjects that concern the glory and honour of his God, is the very person who will be most apt to forget the duties that he owes to his neighbour. The true Christian is characterized not more by his reverential humility than by his gentle philanthropy. The latter grace of character however, to be thoroughly sincere and influential, must be grounded in the former. Sufficient condemnation has been passed upon the oration in question for the outrages it contained against the decencies of social life; we trust that such an exhibition will never be witnessed again in any of our public institutions. There was another blemish, which has not yet, we believe, been noticed, but which (if the above remarks be true) deserves no less a passing reprobation. Among other quotations, intended to illustrate and adorn his speech, Mr. Lawrence adduced those well-known lines from Virgil, as involving a physiological truth:

Principio cœlum, ac terras, camposque liquentes,
Lucentemque globum Lunæ, Titaniaque astra
Spiritus intus alit; totamque infusa per artus
Mens agitat molem, et magno se corpore miscet.
Inde hominum pecudumque genus, vitæque volantum,
Et quæ marmoreo fert monstra sub æquore pontus.

Such a sentiment may be worthy of a heathen poet, a believer in the doctrines of the Pythagorean philosophy that taught that the Deity was nothing more than the *anima* or *spiritus* of the material world, but cannot be received by any one who admits the truth of revelation.

With equal bad taste was the exquisite passage in Lycidas.

So sinks the day-star in the ocean bed,
And yet anon repairs his drooping head,
And tricks his beams, and with new-spangled ore
Flames in the forehead of the morning sky,

(designed, it is well known, to symbolise the resurrection of the redeemed "through the dear might of Him that walked the waves"), forced in at the climax of the peroration to denote or illustrate what?—the resuscitation of Hunter's fame! Were it not for the schoolboy absurdity of the exhibition,—for the speaker suited the action to the word, by waving his hand aloft in repeating the two last lines—we might characterise it as very profanation.

ing organism that re-act on the spiritual principle, this comedy of disease which lays bare all the weaknesses of man, just in the same manner as the 'comedy of death' in olden times sought to shew the vanity of life and of its pomps—it cannot be doubted, we say, but that such instructions daily repeated, and this too not in vain fictions, but in the positive language of actual truths, must tend largely to extinguish that spirit of enthusiasm, that sentiment of the beautiful, which seizes the ideal under the changing forms of sensible reality. If the physician do not guard against such influences as these, by occasionally meditating upon the principles of a philosophy which teaches him to perceive the dignity of human nature, under the mists and clouds that conceal it, he will fail to feel for his brother that tender sympathy which so powerfully seconds the action of the plant of science, and assures its healing virtues against every suffering. The man, who will enter upon the study of medical pursuits without having this antidote, this precious preservative in his head and in his heart, may indeed be able to conquer all the difficulties of the science, but he will soon find the most noble instincts of his soul begin to decay; and, as all the faculties are bound together and give mutual support to each other, the intellect itself will in the long run be crippled in its powers. One of the most constant effects of these studies, pursued without precaution, is to engender a spirit of impudent scepticism; and, along with this vice, there is too often an indulgence in language that is marked not only by levity, but by coarse and disgusting indecency."

After such remarks as these, the reader will not be surprised to hear that Dr. Simon advocates strongly the necessity of a liberal and enlightened education to the young physician, before he enters upon the study of medical pursuits. The importance of this is apt, in the present day, to be too much merged and lost sight of in the unbounded attention that is paid to all the intricacies and minute details of physical and chemical science. The mind should be trained to habits of calm and comprehensive reflection, and taught to remember that the investigation of living and sentient bodies, acted upon too by the various emotions of mind, is not to be pursued upon exactly the same plan that is applicable to the study of brute matter. There is much sound truth in the following estimate of the inductive method of enquiry, applied to medical pursuits.

"The Baconian philosophy—which, by making observation, the observation of phenomena, the only legitimate method applicable to the study of science, has tended so much to foster among medical men the education of the special sensibility of the senses, and has rendered them so adroit in the examination of the symptoms of disease—has doubtless had an injurious effect on the development of the higher faculties of thought and reflection, reducing them often to a sort of scientific incapacity. Although, long before Bacon had invented his method, it is easy to discover a certain number of learned men, and more especially of physicians, who had followed it out in their enquiries, there cannot be a reasonable doubt that the illustrious Chancellor of England, by first clearly laying down the rules for its application, powerfully contributed to popularise it, and that he thereby conferred a very signal benefit on all the physical sciences. Medicine, like the rest, hastened to avail itself of the new method, and to apply its maxims to the investigation of the phenomena of living bodies. And none will deny that, under the guidance of this philosophy, many obscure and perplexing questions have been solved, and that, although men of narrow understanding are continually abusing it by erecting it as the only *organ* of science, (to use an expression of Bacon himself,) much, very much, may yet be achieved by strictly following the laws which it inculcates. We need only point to the recently-opened fields of Organic Chemistry and Microscopic Anatomy as two departments of scientific research, from the careful pursuit of which great benefits may be expected to flow.

"But even without departing from the circle in which the Baconian method confines itself, and exclusively upon the domain of practice, with what accuracy of observation and with what sagacity of comprehension must the medical man be endowed, to seize the sense of every phenomenon on the living tablet of disease, to distinguish the reaction that is sympathetic from the primary morbid movement, and to recognise, under the disguise of different symptoms, the identity of a morbid affection, or to detect the radical difference of the disease under identical symptomatic appearances! It is obvious that it will require much severe mental discipline, before any one can reasonably hope to attain to such intellectual sagacity."

A very common error, among young medical men, is a tendency to exaggerate the truth of the doctrines, and the power of the art, of medicine. Hence the boldness and often dangerous temerity of their practice. How is this evil to be counteracted? Hear what M. Simon says:—"A profound study of the traditions of science, a serious reflection on the causes of the decline and decay of doctrinal systems, which at one time had been received with an almost fanatic enthusiasm, and, above all, a most attentive observation and study of the marvellous resources of Nature to liberate the body from the influence of disease; such are the chief sources from which they will draw that circumspection of conduct, and that prudence in determination, without which the most useful art may become the instrument of irremediable mischief. * * * Prudence is the moral that every well-disciplined mind will draw both from the thoughtful history of the vicissitudes of scientific doctrines, and from the daily lessons of experience; it is the corrective alike of the uncertainties of science, and of the limited capacities of him who applies it to practice."

There cannot be a more dangerous guide in the practice of our profession than the axiom, *melius est progredi per tenebras quam sistere gradum.* The very reverse ought to be the lesson that is taught. Baglivi, with his accustomed wisdom, advises the physician *multa scire, pauca agere.* Valesius remarks, with almost sarcastic force: *periculosius incidere in medicum qui nesciat quiescere, quàm qui nesciat contraria adhibere.* Morgagni uses nearly the same words: *plures sunt medici qui ob id ægros interimunt, quòd nesciunt ipsi quiescere;* and Hoffmann says that, on many occasions, *optimum remedium est nullo uti remedio.* Alas! there are too many in the present day who seem not to appreciate the wisdom of these sentences; men of such extravagant activity that they would take a cudgel to kill a fly! How different has been the conduct of all the best authorities in our profession! Sydenham expressly says: "not only in hysterical affections, but in all diseases, when I do not feel confident that I can do good by my remedies, I think it my wisest part to do nothing at all." In many other passages of his writings, he gives utterance to the same sentiment. Stoll, and indeed every enlightened follower of the Hippocratic school, has followed the same rule.

"In practice they never forgot this inflexible moral precept, *saltem non nocere* —an aphorism, which was inculcated two thousand years ago, by the Coan sage, in words which it cannot be out of place to recall here: περι τα νουσηματα, δυω: ωφελεειν, ἢ μη βλαπτειν, *i. e.* concerning diseases, there are two things: do good, or do no harm. Listen also to the comments of Galen upon this point: it is such an important question, that we cannot accumulate too many authorities to throw light upon it. 'There was a time,' says the illustrious physician of Pergamos, 'when I regarded this precept as of little importance, and as unworthy of Hippocrates. It appeared to me evident that the duty of the physician was to try by all means to relieve his patient. But, after having seen many celebrated physicians justly blamed for the treatment they had pursued, whether by bleeding, or by ordering baths, purgatives, wine or cold water (which became injurious), I then understood that Hippocrates, like many other practitioners of his time, had experienced similar mistakes, and that I should henceforth take all measures, when required to prescribe an important remedy, to calculate beforehand not only what relief the patient might derive if this remedy effected its end, but what harm he might sustain, if it failed. I have never therefore administered any thing, without having taken care not to injure the patient, if I failed in my object. Some physicians, like those who throw the dice, prescribe remedies which are very prejudicial to the patients, if they fail in their object. Those who are beginning the study of our art, will think, I know, as I also thought, that the above precept is not worthy of Hippocrates; but experienced practitioners, I am quite sure, will comprehend all its importance; and, if ever they should happen to injure their patients by the unskilful administration of some powerful remedy, then, above all, will they feel the value of the counsel which Hippocrates has left them.' The precept of Hippocrates, and the reflection of Galen, adds M. Daremberg, who has translated this passage, will find more than one application in our days; there are unhappily many physicians, to whom the patient is but a subject of experiment, for which science is the pretext, but whose real end is too often only personal interest."

We have already seen how earnestly our benevolent author inculcates upon medical men the paramount duty of cultivating a spirit of kindness and charity towards all, with whom they are brought in contact. We shall make no apology for introducing some of his reflections to the notice of our readers.

"While the rich man finds always around him friendly hearts whose sympathy alleviates his sufferings, to the poor man the physician is often the only person before whom he can pour out his grief. Let him at least believe that its expression reaches a heart which understands it. Even hospital physicians owe this token of sympathy to the unfortunate beings whom public charity commits to their care. We are not ignorant that the very considerable number of patients under their charge places them under the cruel necessity of giving to each too limited a time, for it to be devoted to other attentions than those which the chief object of art rigorously imposes upon them. When a legitimate scientific curiosity leads them to be a little less sparing of their time for some of these unfortunate beings, let them at least lend a benevolent attention to the emphatic eloquence of suffering. This duty is the more imperative, as it is the only means of concealing from their eyes that scientific curiosity, to which they scarcely ever submit but with repugnance. 'It is thus,' says Vicq d'Azyr, 'that Lorry became the very comforter of those unfortunates, who, generally without parents, without friends, are disposed to receive even curiosity for interest, when it is accompanied by commiseration.' Yes, mingle some expressions of sympathy with the questions you address to them in the abstract interest of science: you will easily deceive them, and may not a little console them. That is so easy, and may do so much good to these poor creatures, whose sufferings even become a source of instruction, which science turns to the advantage of the more fortunate part of mankind.

"Out of the hospitals, the sacred mission of distributing the aids of science to the poor devolves upon the young physicians. If they should draw from thence their first lessons of experience, who does not see that this intercourse with the unfortunate becomes, at the same time, a touching initiation in the virtues which they ought to practise?* There they may learn pity, disinterestedness, devotedness, in a word, all the virtues which they should bring to the practice of their benevolent art. May this precious education not be fruitless to them; may it develop in their souls that feeling of respect and tender commiseration which they should ever bear towards misfortune! Fothergill, after he had become one of the most successful physicians in England, never forgot, amidst the palaces of the aristocracy, those unhappy beings who had opened to him the path of fame. His heart reverted towards them, as to the source from whence he had drawn those qualities which had secured his prosperity. How many physicians, after the example of Hecquet, Andry, and others, have devoted the last years of their life to the treatment of the poor! thus shewing us that the sweetest recompence the physician can obtain is the sentiment left in the soul by attention to misfortune."

Many indeed are the cases which baffle all the resources of professional skill, and in which it is the painful—but let it not be said, the useless or unprofitable—part of the medical man, day by day, to watch the slow but sure and inevitable advances of a fatal, and it may be a painful, disease. It is the contemplation of such cases as these that has suggested the following beautiful reflections:—

"It is above all when the physician encounters one of those numerous affections which make the *desespoir* of the art, one of those chronic diseases to which he can only oppose a palliative treatment, that it is required of him to supply the insufficiency of science by all the resources of an ingenious charity. Quintilian, who sought to impugn the utility of the medical art by arguments borrowed from the doctrine of fatalism, says, in some part of his writings: '*Fato vivimus, languemus, morimur; medicina quid præstas, nisi juxtà te nemo desperet?*' We accept this judgment in part. Yes, even when the disease has passed the limits within which active medicine is obliged to confine itself, the physician may still be eminently useful to the unhappy beings whom the incurability of disease condemns to certain death. His kind and affectionate words will find, even to the close of the scene, the way that leads to the anguished heart; they will cherish hope; and perhaps even revive, in some cases, the lamp of life which was nearly extinguished. *Animi consolatio, quâcumque causâ fiat, aperit meatus et largam*

---

* In the following sentence, the very same illustration that was applied by Mr. Travers, if we mistake not, to the character of the late Sir Astley Cooper (vide his life by Mr. B. Cooper) as a hospital surgeon, is made use of by our author.

"The habitual spectacle of pain will be apt to extinguish, in the heart of a materialist physician, the tender sympathy which the sight of suffering so naturally kindles in the bosom of others, and to lead him by degrees to look upon his patients only *as the florist looks upon a bed of tulips.*"

Such conduct will have its reward. The time will come when this botanic sort of interest shall cease; and then the mind, if it has no other inward satisfaction save that arising from successful ambition, will waste its waning powers, as old age creeps on, in disappointment and ennui. Far be it from us to accuse the departed of materialism and infidelity. Still we much fear that neither our distinguished countryman, nor his equally celebrated continental rival of the French metropolis, was actuated by the pure and lofty principles inculcated by our author. Both died disappointed men.

*perspirationem facit.* There is not a single physician, we are certain, however inattentive he may be to the progress of chronic affections, who has not had to observe the soothing influence exercised upon the general state of invalids by that disposition of mind alluded to by the celebrated professor of Padua, in the aphorism we have just quoted. But it is above all the physician who can handle with success the instrument of this moral treatment; it is above all his friendly sympathising speech, which can shed hope upon this troubled soul. How much then must it be his imperative duty to bring to the practice of the art those moral qualities which ought, if I may so speak, enlarge and extend its limits."

That the inordinate and almost exclusive attention, which has, for the last forty years more especially, been paid to the physical effects of—or, in other words, the anatomical lesions left by—disease, has tended to materialise the intellectual character of medical men, we have already had occasion to maintain. That it is apt, at the same time, to deaden the sensibility, and check the outgoings of sympathy from the heart, will be admitted by many as well as by M. Simon.

"It is more especially since the study of Pathological Anatomy has introduced its important data into the science of Medicine, that the compassion of the physician for the sufferings of human nature has ceased to be perceived in works descriptive of disease, and that it no longer gives a colouring to the sad details of suffering and death. There, the heart never breathes any warmth into science; there, sentiment never beams upon the dark picture of the miseries of life. Whether it treats of a disease which, like pulmonary phthisis, seems to select its victims from among those into whose minds the hand of God has poured his most precious gifts, or of that terrible affection which strikes the mother in the midst of the sweet delights of maternity, and deprives the infant at its birth of that heart in which it should pass the first years of the second phasis of its life, as the first was passed in the cradle of flesh where it was conceived; in a word, in diseases too numerous to be named, where art can only deplore its impotency, as well as in those in which it displays all its efficacy, there is nothing which proceeds from the heart, there is nothing which evidences the sympathy of the physician for the sufferings of humanity; it would seem as if this humanity were become simply an object of natural history;—*absentem, marmoreamve putes.* There are even some of these works where not only you would search in vain for a reflection of that human sympathy which ought so naturally to arise in the heart of the physician, but where, in place of this sentiment, you will observe a kind of ill-dissembled joy, in the prospect of the fatal event which gives the opportunity of verifying an uncertain diagnosis."

Alas! is there not too much truth in the bitter reproof which these last words convey? It cannot be denied. The satisfaction at finding one's professional predictions confirmed by dissection, too often, we fear, sears the heart against any tender emotions which might otherwise have arisen.

We are glad to find that Dr. Simon, while he does not absolutely condemn the performance of experiments upon living animals, denounces with great force and truth that revolting spirit of reckless disregard of suffering which has characterised not a few of the physiologists of the present day. Even he however is, we think, far too tender to such men as Majendie and Flourens, in excusing these barbarities on the score of the "remarquables enseignements" which they have taught us. We attach infinitely less value to the vaunted discoveries of these experimenters than he and perhaps most medical men seem willing to admit; and for this very simple reason: have they taught us better than we knew before how to prevent and cure disease, or to mitigate suffering, or to prolong and sweeten life? Oh no; but then they have solved some perplexing physiological problem, or corrected some prevailing physiological error, or established some grand physiological doctrine! Truly has our author said that, "before any one can acquire that special aptitude to discover truth amidst the proteiform reactions of struggling sensibility, one needs to have the placidity of a butcher, or of one of the knackers of Montfauçon."

Even to the dead relics of humanity, it is right and proper that some shew of respect, some degree of religious decorum, be paid. It is quite possible to associate this sentiment of regard with the exigencies of science. There is no doubt but that he, who thinks of the change which that pale corpse is yet destined to undergo, will cease to view it as mere brute matter to be experimented upon. When a more generous philanthropy shall animate the zeal of medical men in behalf of their suffering brethren, they will be naturally led to view their mortal remains with a feeling almost akin to religious respect. Might it not be well to promote and encourage this feeling by inscribing on the walls of the dead-house and dissecting-room a moral sentence, a hallowed text here and there, to recall the frivolous or irreverent mind to thoughtful meditation? Think you that science would lose aught of what might be truly useful, if its pursuit was blended with an occasional solemn reflection? No, it would only become more sanctified and ennobled. A remark of Victor Hugo may be justly applied to this subject. "Through all things," says this distinguished writer, "see that a moral and sympathetic thought is made to circulate, and then there will be nothing deformed or repulsive. Associate a religious idea with an object the most hideous, and straightway it will become pure and holy. Affix God to a gibbet, and you have the cross."

It would have given us great pleasure to have followed our author in the examination of many of the other chapters of his work. Our object, however, has been rather to give our readers an idea of its general tone and bearings, than to analyse it in detail. It might, indeed, have been not without use in the present day, when there is so much laxity of professional decorum if not of professional principle also, to have considered some of the special duties of medical men to one another, as well as to the public. Want of space, however, entirely precludes us from doing so now; but we regret this omission the less, as the great object in every enquiry upon ethics and morality should be to trace them upwards to the source from whence they flow, the only sure foundation on which they can be built—Religion. If the spring be pure, the stream will be pure also; make the tree good, and its fruit will be good also.

There is, however, one chapter to which, as treating of our own gentle craft,—"De la critique en medecine"—we must afford a brief and passing notice. A single extract will suffice:—

"If all medical writers were influenced solely by the love of truth and by the sincere desire to benefit their fellow-creatures, the task of the critic would be alike an easy and a pleasant one. But unfortunately such is not the case, and not a month passes over our heads that we do not meet with some lamentable instance or another of medical works, that are the offspring of very different motives. Such publications, as we are now alluding to, might be passed over, in other departments of literature or science, as utterly contemptible and unworthy of notice. In medicine, however, it is otherwise; for even the meanest of them is evidence in itself of suffering and mischief that have already been inflicted. Is it not therefore to be desired by all who have the good of their profession, as well as the public advantage, at heart, that there should be some controuling power to check the spread and counteract the influence of the many flimsy and misleading works, that are continually issuing from the press? The difficulty of correct observation, the natural tendency of the mind to find out between facts a relation which an unprejudiced person will utterly fail to perceive, and the constant operation of those active vital forces which are invariably involved in the production of disease, and which continually serve to disguise the influence of the curative means employed—such are (some of) the principal causes which render it necessary to have the controul of the critic upon medical works. Some one will probably say, how is he (the critic) to judge of the value of many new proposals, before he has had an opportunity of testing them by actual experience? The argument is more specious than solid. How many *new* proposals are but the iteration of what has already been tried, and found wanting! And how often do the very facts, adduced in support of their claims, but serve to disclose most convincingly their fallacy and worthlessness! It is, moreover, always to be borne in mind that, when we talk of experience in medical matters, the trial has to be made upon a human being, who applies to us for relief, and is not to be regarded as brute matter on which we may experiment at our will."

Whatever be the subject under discussion, M. Simon treats it with high and elevated views. Every thing appertaining to the life of man ought, in his opinion, to have an air of seriousness cast around it. We think that, in the main, he is quite right. The medicine of the present day has too little spirituality in it; it savours too much of materialism. It is dissociated from some of its highest privileges, from its noblest ambition. It has mixed itself up with the mercantile spirit of the age; and its aims and ends have necessarily become secular and worldly. As long as this remains the character of our profession, it can never occupy the position in the social scale which it ought to do. It will be regarded as a highly useful art, but not as a dignified and dignifying science.

If medical men think fit to accept the eulogy of the Roman orator (quoted at the beginning of this article) upon their profession, let them remember that just in proportion to its claims to dignity and respect, so is the amount of its responsibilities and duties. Let them not suppose that these are summed up in the skilful administration of remedies, or in the dexterous performance of operations. Let them recollect that the being, whom they undertake to relieve, has susceptible moral feelings, and a thinking, reasoning, soul, as well as a sentient and impressionable body; and that that body and that soul are so intimately, although mysteriously, bound together, and so mutually dependent upon each other in this state of existence, that the one cannot suffer without the other sympathising and being acted upon. Let them think of the fact that many corporeal diseases are aggravated, or even induced, by a feverish or perturbed state of mind; while, on the other hand, some of the sorest ills, that flesh is heir to, are more soothed by patient resignation and animating hope than by "all the drowsy syrups of the world." Let them bear in mind that every affliction, bodily and mental, is no blind accident or chance event; but has been sent in mercy, and is designed for some gracious end, and may therefore become the instrument either of peace and happiness or of misery and condemnation to him who is tried thereby; that every sufferer whom they visit is hastening on to another world, and that every death which they behold, while it closes the pilgrimage on earth, is the commencement either of eternal joy or of everlasting woe. When they think of these solemn things, and remember at the same time that to all are the offers of peace and acceptance made; that all are invited to partake of the heavenly blessing; that there is still a fountain open whose waters have the power to heal, and that still a virtue goes out from Him, the very touch of whose garment sufficed to give health and vigour to her who had spent all her substance upon the physicians in vain—shall they, who have taken upon themselves the sacred mission of ministering to the sick, not have one word of comfort or of kind admonition to offer to the poor sufferer, trembling on the very verge of dissolution, and who may have no other ear to listen to his prayers and sorrows save that of his physician? Do they not know how much the heart is softened by sickness, and how often the spirit, which has been hitherto thoughtless or insensate, is subdued under the chastisement of pain?—that the ground, thus prepared, is ready for the good seed, if dropped into it; that a word in season may become the message of glad tidings to the soul; that a blessing is promised alike to him who gives and to him who receives; that the apprehension of alarming the fears, and thus of aggravating the danger of an existing disease, can never be a justification for neglecting so hallowed a duty; that, if the opportunity be lost, the hour of repentance and consolation may be for ever gone: for as the tree falleth, so must it lie; as death leaves the man, eternity shall find him. It is impossible for the physician to shut out these thoughts entirely from his mind; they will obtrude and force themselves upon his reflection, whether he will or not. A death-bed is too solemn a sight even to him, not to stir up, every now and then, a train of solemn meditation. Let him not wish to stifle or evade its appeal to his heart; let him rather seek to profit by the lesson which it conveys. We have been told that—

> "An undevout astronomer is mad:"

What shall we say of an irreligious physician? The one has but to do with the marvels of the material universe, great and glorious as this is; the other is called upon not only to behold the fearful and wonderful mechanism of living sentient nature, but also to deal with the welfare and happiness of immortal men.

